# Overuse of Cervical Cancer Screening Tests Among Women With Average Risk in the United States From 2013 to 2014

**DOI:** 10.1001/jamanetworkopen.2021.8373

**Published:** 2021-04-29

**Authors:** Jason D. Wright, Ling Chen, Ana I. Tergas, Alexander Melamed, Caryn M. St. Clair, June Y. Hou, Fady Khoury-Collado, Allison Gockley, Melissa Accordino, Dawn L. Hershman

**Affiliations:** 1 Columbia University College of Physicians and Surgeons, New York, New York; 2 Herbert Irving Comprehensive Cancer Center, New York, New York; 3 New York Presbyterian Hospital, New York, New York; 4 Joseph L. Mailman School of Public Health, Columbia University, New York, New York

## Abstract

**Importance:**

While cervical cancer screening with cytologic and human papillomavirus (HPV) testing has reduced mortality from cervical cancer, overuse of these tests is associated with downstream psychological and medical consequences, as well as significant costs. Guidelines now recommend less frequent testing, although adherence to these recommendations is uncertain.

**Objective:**

To determine the frequency of overuse of cervical cancer screening tests.

**Design, Setting, and Participants:**

This cohort study included 2 299 177 women aged 30 to 65 years recorded in the MarketScan database who underwent cervical cancer screening with cervical cytologic testing, cotesting, or primary HPV testing in 2013 through 2014. Women were followed-up for 3 years, and use of repeat testing during this period was noted. Clinical and demographic characteristics associated with overuse of screening and the association between screening and performance of routine gynecologic examinations were recorded. Data were analyzed from June 15 to September 15, 2020.

**Main Outcomes and Measures:**

Cumulative performance of overuse of cervical cancer screening with repeat cytologic or HPV testing within 36 months of the index screening test.

**Results:**

A total of 2 299 177 women with a median (interquartile range) age of 47 (39-54) years were identified. Initial cervical cancer screening consisted of cytologic testing alone in 1 286 179 women (55.9%), cotesting in 991 583 women (43.1%) and HPV testing in 21 415 women (0.9%). The cumulative incidence of repeat cervical cancer screening was 17.7% (95% CI, 17.6%-17.7%) at 12 months, 51.1% (95% CI, 51.0%-51.2%) at 24 months and 65.8% (65.7%-65.8%) at 36 months. Repeat screening was less common in older women (32 198 women [60.2%] aged 60-64 years vs 194 665 women [67.8%] aged 30-39 years; *P* < .001), women with medical comorbidities (125 197 women [64.1%] with ≥2 comorbidities vs 423 012 women [64.7%] with no comorbidities; *P* < .001), women screened in 2014 (176 734 women [53.4%] in 2014 vs 555 767 women [69.4%] in 2013; *P* < .001), and those screened with cotesting (277 032 women [56.9%] for cotesting vs 450 438 [71.2%] for cytologic testing; *P* < .001). In contrast, overuse of testing was more common in the Northeastern US (143 916 women [68.1%] in the Northeast vs 81 552 women [51.6%] in the West; *P* < .001), in women with more frequent outpatient visits (238 510 women [71.1%] with ≥6 visits vs 279 412 [58.7%] with ≤2 visits; *P* < .001). Women with a sexually transmitted infection after their index testing were also more likely to undergo repeat testing (adjusted odds ratio, 1.42 [95% CI, 1.21-1.68]). Women who did not undergo repeat screening were significantly less likely to undergo a gynecological exam after the index screening test: during year 2 of follow-up, 657 749 women (96.7%) who underwent repeating screening had a gynecological examination compared with 203 566 women (26.2%) who did not undergo a gynecological examination.

**Conclusions and Relevance:**

These findings suggest that among commercially insured women with average risk, cervical cancer screening tests were frequently overused.

## Key Points

**Question:**

How often are cervical cancer screening tests overused in women with average risk in the United States?

**Findings:**

In this cohort study of 2 299 177 commercially insured women aged 30 to 65 years who underwent cervical cancer screening in 2013 through 2014, 64.7% underwent repeat testing within 36 months of index testing.

**Meaning:**

These findings suggest that despite evidence-based guidelines, overuse of cervical cancer screening tests was common.

## Teaser

This cohort study examines the frequency of overuse of cervical cancer screening tests in women with average risk in the United States.

## Introduction

The introduction of organized cervical cancer screening programs has reduced the mortality of cervical cancer.^1,2^ Screening has historically relied on cytologic examination of exfoliated cells from the cervix, known as the Papanicolaou test. Recognition of the causal association between human papillomavirus (HPV) infection and cervical cancer has led to the incorporation of HPV testing into screening paradigms.^2^ While HPV testing was initially used in combination with cytologic testing, so called *cotesting*, more recently, primary HPV testing alone has also been endorsed as a screening strategy.^3^

The 2012 American Cancer Society guidelines for cervical cancer screening recommend use of cytologic screening every 3 years or cotesting with HPV and cytologic testing every 5 years in women aged 30 to 65 years.^2^ More recently, guidelines have been updated to advocate for primary HPV testing every 5 years as the preferred approach to screening in all women aged 25 to 65 years.^3^ These evidence-based recommendations attempt to balance cervical cancer prevention with the potential harms of screening.^2,3^ As many episodes of HPV infection and low-grade dysplasia are transient, identification of these abnormal findings increases use of colposcopy and follow-up testing, may lead to patient anxiety and psychological distress, and substantially increases health care costs.^4^ Modeling studies indicate that performing screening tests more frequently than every 3 years for cytologic examination or every 5 years for HPV-based testing is associated with significantly increased rates of colposcopy with little association with the incidence of cervical cancer.^2,5,6,7,8^

Despite the potential harms of overscreening, patient desire for more frequent testing, inconsistent recommendations for screening by clinicians, and lack of familiarity with guidelines among clinicians are common.^9,10^ Prior studies of overuse of screening have predominately focused on testing young women, elderly women, and those who have undergone hysterectomy, and these reports have identified widespread variation in screening patterns and use of testing.^11,12,13,14^ The objective of our study was to examine the patterns of cervical cancer screening among average risk women in the US. Specifically, we examined the frequency of testing and follow-up for women with cervical cytologic testing, cotesting, and primary HPV testing among women aged 30 to 65 years.

## Methods

### Data Source

Data for this cohort study were deidentified and deemed non–human participant research by the Columbia University institutional review board; therefore, it was exempt from approval and informed consent. This study is reported following the Strengthening the Reporting of Observational Studies in Epidemiology (STROBE) reporting guideline for cohort studies.

Data for this study were collected from the IBM Watson Health MarketScan database. MarketScan includes medical claims for inpatient and outpatient services and outpatient pharmaceutical claims from more than 50 million privately insured individuals nationwide.^15^ Data undergo multiple checks for validity and quality control from initial case collection to final data output.^15^

### Cohort Selection and Classification

We identified women aged 30 to 64 years who had a claim for cervical cytologic testing, cotesting, or primary HPV testing in 2013 through 2014. Screening recommendations during this time recommended cytologic testing every 3 years or cotesting with HPV and cytologic examination every 5 years in women aged 30 to 65 years.^2^ Claims for cytologic and HPV testing that occurred within 30 days were considered cotesting. Claims that occurred within 60 days from one another were considered 1 test, and the date of the earliest claim was used as the test date, whereas claims that occurred more than 60 days apart were considered different tests. For women who had more than 1 test in the 2-year period, the earliest test was chosen as their index test.

We excluded women who did not have continuous insurance coverage for 12 months prior to and 12 months after the index test. To exclude women with underlying cervical dysplasia, we excluded women whose tests had abnormal cervical cytologic or histologic findings or who underwent gynecological procedures for the evaluation or treatment of cervical disease, including colposcopy, biopsy, excision, and cauterization, during the period 12 months prior to or 12 months after the index procedure. To capture women with average of cervical cancer, we excluded women for whom more frequent testing is considered appropriate, including women with diethylstilbestrol exposure, HIV infection, leukemia, neutropenia, or organ transplantation or who used immunosuppressants at any time (eTable 1 in the Supplement). Women who underwent hysterectomy and those with gynecological cancers were excluded. Women were followed-up for up to 3 years after their index test.

### Demographic and Clinical Characteristics

Demographic and clinical characteristics included age (categorized as 30-39, 40-49, 50-59, and 60-64 years), year of the index test (2013 or 2014), type of index test (ie, cotesting, cytologic testing, or primary HPV testing), residence in a metropolitan statistical area (MSA; categorized as MSA, non-MSA, or unknown), and region (ie, Northeast, North central, South, West, or unknown). Medical history, including comorbidity, mental health or substance abuse disorders, sexually transmitted diseases, gynecological symptoms, and pregnancy, were evaluated using claims that occurred within 12 months before the index test.^16^ Comorbidity was measured using the Elixhauser comorbidity index and classified as 0, 1, or 2 or more comorbidities, excluding the comorbid conditions of mental health or substance abuse disorders.^17^ Mental health and substance abuse disorders were each analyzed and recorded if 2 claims that were more than 30 days apart were identified. A prior study examining overuse of cervical cancer screening reported decreased testing among women with mental health and substance abuse disorders.^16^ We hypothesized that greater interaction with the health care system may increase the chance of additional cervical cancer screening. As previously described, we examined medical service utilization potentially related to cervical cancer screening, including the annual number of outpatient visits, family planning services within 30 days prior to or after the index test, and sexually transmitted disease testing in the 12-month period after the index test.^16^ The number of outpatient visits were estimated by counting the number of claims from outpatient clinic visits that were more than 7 days apart, and classified as 0 to 2, 3 to 5, and 6 or more visits.^16^

### Statistical Analysis

The primary analysis used a Kaplan-Meier time to event analysis. Women were followed up for up to 36 months and considered to have had an event when a code for cervical cytologic testing, cotesting, or an HPV test at least 60 days after the index test were recorded. Patients were censored at the time of a lapse of insurance coverage. To women with testing performed for the evaluation of an abnormal finding, women were censored at the time of a billing claim for any abnormal cervical or vaginal cytologic or histologic finding, an unsatisfactory Papanicolaou test finding, a positive results in an HPV test, or if they had a code for a colposcopy, biopsy, excisional procedure, or cauterization of the cervix. The entire cohort was analyzed, and a second analysis was performed stratified based on the type of index test.

**Figure 1.**
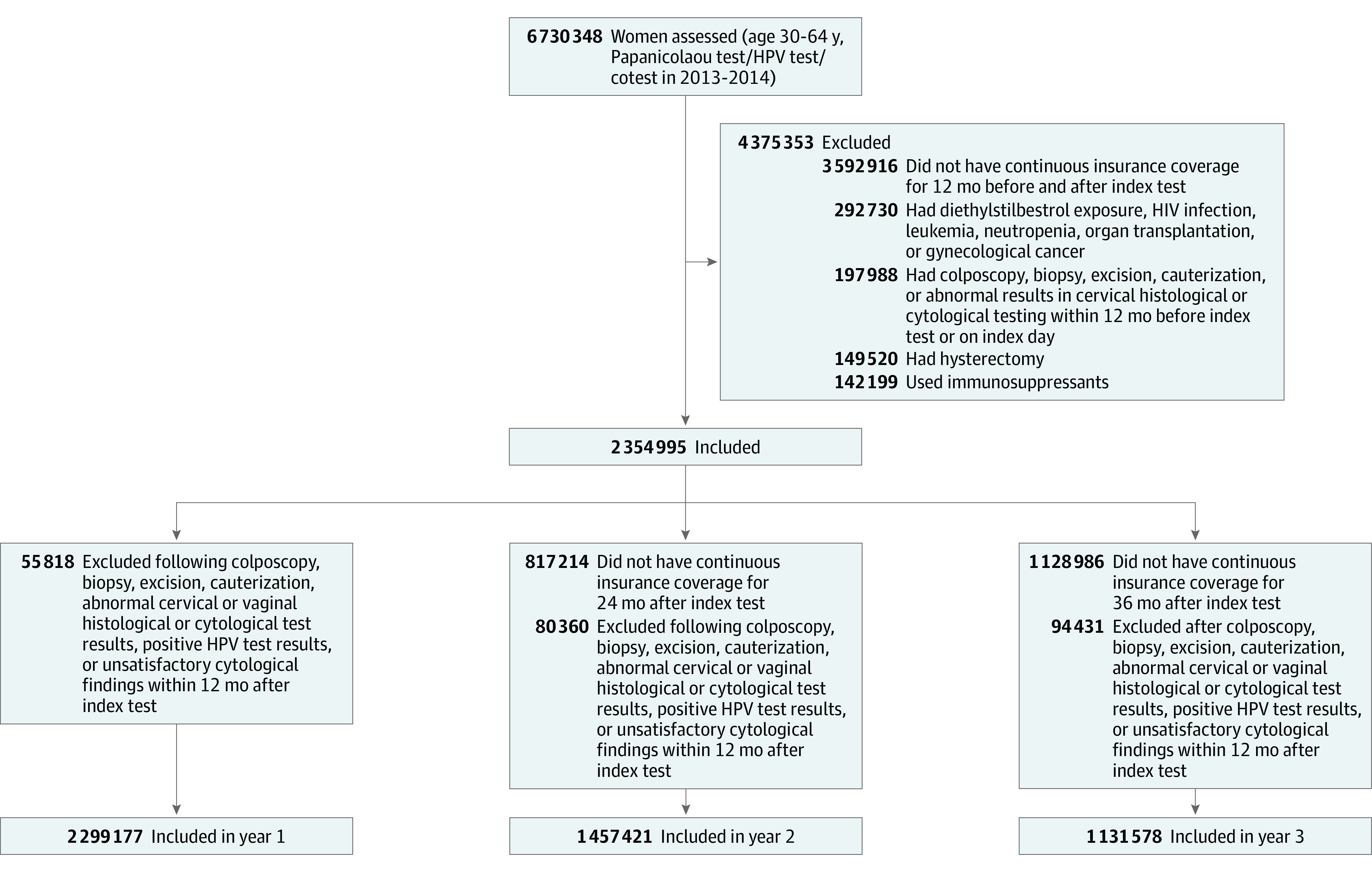
Flowchart of Cohort Selection HPV indicates human papillomavirus.

To descriptively determine the characteristics of women with downstream testing as well as use of gynecological examinations during follow-up, testing patterns during years 2 and 3 of follow-up were examined. Similar to the Kaplan-Meier analysis, to exclude women who underwent repeat testing for the evaluation of an abnormal finding, women were excluded from a given follow-up period and all subsequent follow-up if they had code for abnormal cervical or vaginal cytologic or histologic findings, an unsatisfactory Papanicolaou test result, positive results in an HPV test, or had a code for colposcopy, biopsy, excision, or cauterization of the cervix during a given follow-up period (Figure 1). Interval tests were classified as cytologic, primary HPV, or cotesting using a similar classification as described for the index test. Women in the year 2 cohort who had a test 12 to 24 months after the index test and those in the year 3 cohort who had a test at 24 to 36 months were considered as having an interval test during the respective periods. The time from the prior test to the interval test was described as medians and interquartile ranges (IQRs).

The demographic and clinical characteristics were reported as number and proportions. Women who had a second test within 36 months of the index test were considered to have had overuse of cervical cancer screening tests. For women with 3 years of follow-up, we compared clinical and demographic characteristics between women with and without overuse of testing using χ^2^ tests. A multivariable logistic regression model was fit to estimates factors associated with overuse of testing, adjusting for all the demographic and clinical characteristics.

We examined the use of gynecological examinations in years 2 and 3 after the index test, and compared the use among women based on performance of interval cervical cancer screening tests. We fit similar multivariable logistic regression models in each cohort for risk factors of gynecological examination.

All analyses were performed using SAS statistical software version 9.4 (SAS Institute). All tests were 2-sided, and a *P* < .05 was considered statistically significant. Data were analyzed from June 15 to -September 15, 2020.

## Results

A total of 2 299 177 women were identified (Figure 1). Initial cervical cancer screening consisted of cytologic testing alone in 1 286 179 women (55.9%), cotesting in 991 583 women (43.1%), and primary HPV testing in 21 415 women (0.9%) (Table 1). The median (IQR) age of the cohort was 47 (39-54) years. A total of 1 301 469 women (56.6%) had no comorbidities, and 574 540 women (25.0%) had 1 comorbidity. Prior to the index screening examination, 136 833 women (6.0%) experienced a gynecological symptom within 30 days of the index test, and 85 753 women (3.7%) had been pregnant in the prior year.

**Table 1.** Clinical and Demographic Characteristics of the Cohort

Characteristic	Initial cohort, No. (%) (N = 2 299 177)	Cohort with 3 y of follow-up			
		No. (%)		*P* value	Multivariable models of testing overuse, aOR (95% CI)
		No overuse of cervical screening tests (n = 399 077)	Overuse of cervical screening tests (n = 732 501)		
Age, y					
30-39	604 358 (26.3)	92 449 (32.2)	194 665 (67.8)	<.001	1 [Reference]
40-49	742 827 (32.3)	129 827 (34.3)	249 088 (65.7)		0.89 (0.88-0.90)^a^
50-59	746 852 (32.5)	155 546 (37.7)	256 550 (62.3)		0.73 (0.72-0.73)^a^
60-64	205 140 (8.9)	21 255 (39.8)	32 198 (60.2)		0.63 (0.62-0.64)^a^
Year of index test					
2013	1 722 141 (74.9)	244 938 (30.6)	555 767 (69.4)	<.001	1 [Reference]
2014	577 036 (25.1)	154 139 (46.6)	176 734 (53.4)		0.61 (0.60-0.61)^a^
Type of index test					
Cotesting	991 583 (43.1)	210 089 (43.1)	277 032 (56.9)	<.001	1 [Reference]
Cytologic	1 286 179 (55.9)	182 479 (28.8)	450 438 (71.2)		1.86 (1.85-1.88)^a^
HPV testing	21 415 (0.9)	6509 (56.4)	5031 (43.6)		0.63 (0.61-0.66)^a^
Metropolitan statistical area					
Yes	1 961 975 (85.3)	347 639 (35.3)	638 320 (64.7)	.58	1 [Reference]
No	290 262 (12.6)	50 255 (35.3)	91 942 (64.7)		0.83 (0.82-0.84)^a^
Unknown	46 940 (2.0)	1183 (34.6)	2239 (65.4)		1.22 (0.86-1.71)
Region					
Northeast	465 283 (20.2)	67 353 (31.9)	143 916 (68.1)	<.001	1 [Reference]
North Central	438 605 (19.1)	87 337 (41.3)	124 166 (58.7)		0.58 (0.57-0.58)^a^
South	973 383 (42.3)	166 705 (30.5)	380 530 (69.5)		0.91 (0.90-0.92)^a^
West	374 680 (16.3)	76 443 (48.4)	81 552 (51.6)		0.47 (0.46-0.48)^a^
Unknown	47 226 (2.1)	1239 (34.6)	2337 (65.4)		0.56 (0.40-0.78)^a^
Comorbidity score					
0	1 301 469 (56.6)	230 388 (35.3)	423 012 (64.7)	<.001	1 [Reference]
1		98 498 (34.8)	184 292 (65.2)		0.94 (0.93-0.95)^a^
≥2	423 168 (18.4)	70 191 (35.9)	125 197 (64.1)		0.85 (0.84-0.86)^a^
Mental health or substance abuse	230 742 (10.0)	37 574 (36.5)	65 380 (63.5)	<.001	0.81 (0.80-0.83)^a^
Outpatient visits within 12 mo prior, No.					
0-2	924 734 (40.2)	196 794 (41.3)	279 412 (58.7)	<.001	1 [Reference]
3-5	653 262 (28.4)	105 142 (32.9)	214 579 (67.1)		1.32 (1.30-1.33)^a^
>6	721 181 (31.4)	97 141 (28.9)	238 510 (71.1)		1.60 (1.59-1.62)^a^
Sexually transmitted disease within 12 mo prior	1856 (0.1)	199 (24.6)	609 (75.4)	<.001	1.42 (1.21-1.68)^a^
Pregnancy within 12 mo prior	85 753 (3.7)	11 819 (29.5)	28 210 (70.5)	<.001	0.95 (0.92-0.97)^a^
Gynecologic symptoms within 30 d of test	136 833 (6.0)	22 771 (35.3)	41 824 (64.7)	.93	0.97 (0.95-0.99)^a^
Sexually transmitted disease testing within 12 mo prior	353 417 (15.4)	56 407 (34.1)	109 193 (65.8)	<.001	1.07 (1.06-1.08)^a^
Family planning within 30 d of test	86 521 (3.8)	14 279 (37.0)	24 335 (63.0)	<.001	0.91 (0.89-0.93)^a^

Abbreviations: aOR, adjusted odds ratio; HPV, human papillomavirus.

^a^ *P* < .05.

**Figure 2.**
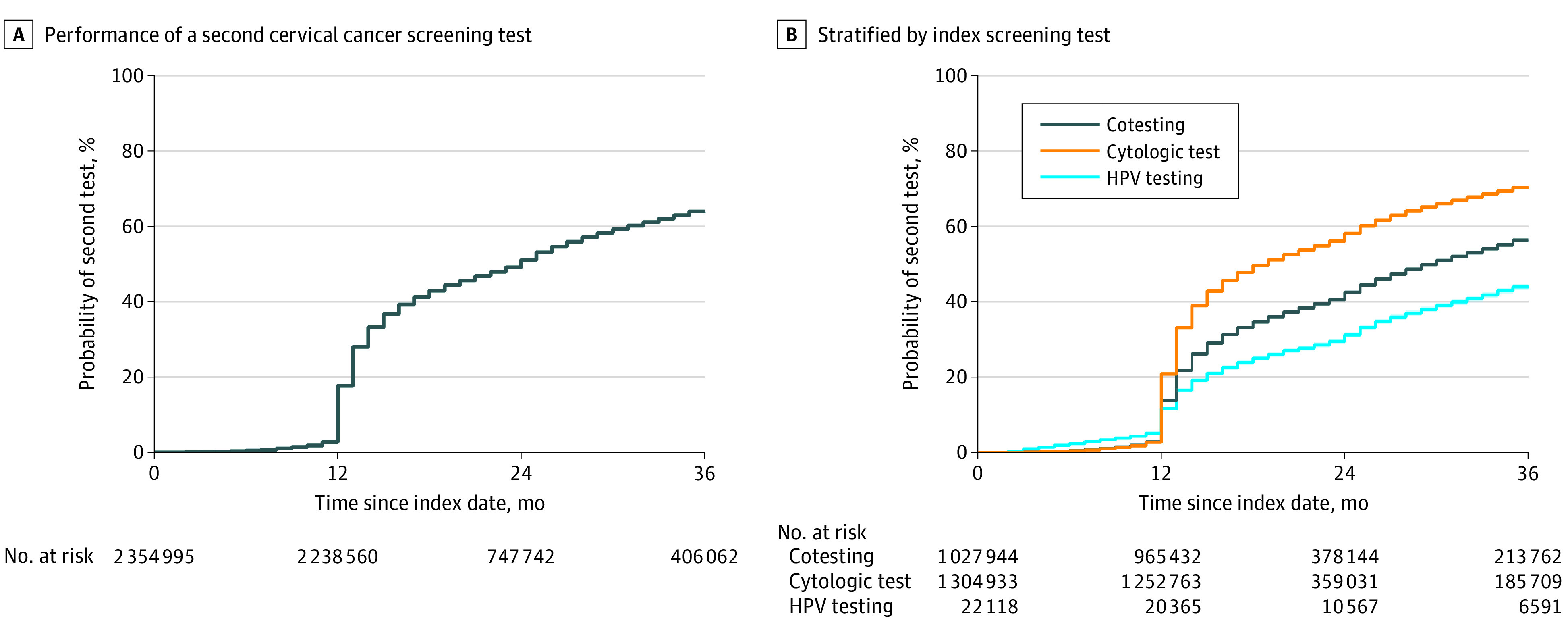
Kaplan-Meier Time to Event Analyses HPV indicates human papillomavirus.

The cumulative incidence of repeat cervical cancer screening was 17.7% (95% CI, 17.6%-17.7%) at 12 months, 51.1% (95% CI, 51.0%-51.2%) at 24 months and 65.8% (95% CI, 65.7%-65.8%) at 36 months (Figure 2A). Compared with women initially screened with cotesting, repeat testing was more common in women initially screened with cytologic testing (adjusted odds ratio [aOR], 1.86; 95% CI, 1.85-1.88) and less common in those screened with primary HPV testing (aOR, 0.63; 95% CI, 0.61-0.66) (Table 1 and Figure 2B). Repeat screening was less common in older women (32 198 women [60.2%] aged 60-64 years vs 194 665 women [67.8%] aged 30-39 years; *P* < .001), patients with medical comorbidities (125 197 women [64.1%] with ≥2 comorbidities vs 423 012 women [64.7%] with no comorbidities; *P* < .001), women screened in 2014 (176 734 women [53.4%] in 2014 vs 555 767 women [69.4%] in 2013; *P* < .001), and those screened with cotesting (277 032 women [56.9%] for cotesting vs 450 438 [71.2%] for cytologic testing; *P* < .001) (Table 1). Additionally, women who were pregnant, had gynecologic symptoms, patients with mental health or substance abuse, or used family planning services around the index test were less likely to undergo repeat screening (Table 1). In contrast, overuse of testing was more common in the Northeastern US (143 916 women [68.1%] in the Northeast vs 81 552 women [51.6%] in the West; *P* < .001). Patients with an sexually transmitted infection after their index test were also more likely to undergo repeat testing (adjusted odds ratio, 1.42 [95% CI, 1.21-1.68]) (Table 1).

Overall, 680 029 women (46.7%) underwent a repeat screening test in year 2 (12-24 months after the index test) (Table 2). Among women who had a second test 2 to 12 months after the index test, 22 057 (35.3%) had a repeat screening test 12 to 24 months after the index screen, while 657 972 women (47.2%) without a screening test 2 to 12 months after the initial screening underwent repeat testing at 12 to 24 months. The median (IQR) time between the year 2 test and prior screening evaluation was 13.4 (12.4-16.2) months. Among patients who had a repeat test in year 2, cytologic examination was most common (393 817 women [57.9%]), followed by cotesting (281 495 women [41.4%]) and primary HPV testing (4717 women [0.7%]) (Table 2).

**Table 2.** Performance of Repeat Cervical Cancer Screening After Index Testing

	No. (%)	
	Year 2 (n = 1 457 421)	Year 3 (n = 1 131 578)
Interval test during given follow-up period, No. (%)	680 029 (46.7)	507 900 (44.9)
Time from last test before the interval, median (IQR), mo	13.4 (12.4-16.2)	14.5 (12.6-26.0)
Type of interval test, No. (%)		
Cotesting	281 495 (41.4)	242 585 (47.8)
Cytologic	393 817 (57.9)	261 328 (51.5)
HPV testing	4717 (0.7)	3987 (0.8)
Patients with a test in the prior 12 mos, No.	62 558	534 089
Interval test in year 2 or 3, No. (%)	22 057 (35.3)	323 693 (60.6)
Time from last test, median (IQR), mo	12.2 (11.4-12.6)	12.8 (12.4-14.4)
Patients without a test in the prior 12 mo, No.	1 394 863	597 489
Interval test in year 2 or 3, No. (%)	657 972 (47.2)	184 207 (30.8)
Time from last test, median (IQR), mo	13.5 (12.5-16.4)	28.3 (25.4-32.2)

Abbreviations: HPV, human papillomavirus; IQR, interquartile range.

A total of 507 900 women (44.9%) received a repeat screening test in year 3 (24 to 36 months after the index test) (Table 2). Repeat screening was performed in 323 693 women (60.6%) who had been screened in year 2 and in 184 207 women (30.8%) who had not had screening during the second year of follow-up (Table 2). The median (IQR) time between the year 3 test and the prior test was 14.5 (12.6-26.0) months. Screening during the third year of follow-up was cytologic in 261 328 women (51.5%), cotesting in 242 585 women (47.8%), and HPV testing in 3987 women (0.8%). The rate of repeat screening decreased with age, with 3-year repeat screening occurring in 194 665 women (67.8%) aged 30 to 39 years, 249 088 women (65.7%) aged 40 to 49 years, 256 550 women (62.3%) aged 50 to 59 years, and 32 198 women (60.2%) aged 60 to 64 years (eTable 2 in the Supplement).

A billing code for a gynecological examination during year 2 was reported in 657 749 women (96.7%) who underwent interval screening, compared with 203 566 women (26.2%) who did not undergo cervical cancer screening that year (aOR, 0.01; 95% CI, 0.01-0.01; *P* < .001) (Table 3). During year 3, a billed gynecologic examination was noted in 490 121 women (96.5%) who had a cervical cancer screening test compared with 142 578 women (22.9%) who did not have testing (aOR, 0.01; 95% CI, 0.01-0.01; *P* < .001).

**Table 3.** Performance of Gynecologic Examinations After Index Cervical Cancer Screening

Patient testing status and characteristics	Year 2^a^	Year 3^a^
**Patients undergoing examination, No. %**		
Patients who underwent an interval test during given year		
Gynecologic examination	657 749 (96.7)	490 121 (96.5)
No gynecologic examination	22 280 (3.3)	17 779 (3.5)
Patients who did not undergo an interval test during given year		
Gynecologic examination	203 566 (26.2)	142 578 (22.9)
No gynecologic examination	573 826 (73.8)	481 100 (77.1)
*P* value	<.001	<.001
**Factors associated with of gynecological examination, aOR (95% CI)**		
Interval test during given period		
Yes	1 [Reference]	1 [Reference]
No	0.01 (0.01-0.01)^b^	0.01 (0.01-0.01)^b^
Age, y		
30-39	1 [Reference]	1 [Reference]
40-49	1.06 (1.05-1.08)^b^	1.05 (1.04-1.07)^b^
50-59	0.97 (0.96-0.98)^b^	0.96 (0.94-0.97)^b^
60-64	0.91 (0.89-0.93)^b^	0.89 (0.87-0.92)^b^
Year of index test		
2013	1 [Reference]	1 [Reference]
2014	0.87 (0.86-0.88)^b^	0.80 (0.79-0.81)^b^
Type of index test		
Cotesting	1 [Reference]	1 [Reference]
Cytologic	0.505 (0.500-0.510)^b^	0.69 (0.68-0.70)^b^
HPV	1.12 (1.07-1.16)^b^	1.07 (1.02-1.13)^b^
Metropolitan statistical area		
Yes	1 [Reference]	1 [Reference]
No	1.06 (1.04-1.07)^b^	1.05 (1.04-1.07)^b^
Unknown	6.01 (3.74-9.66)^b^	4.56 (2.59-8.05)^b^
Region		
Northeast	1 [Reference]	1 [Reference]
North Central	0.91 (0.90-0.93)^b^	0.96 (0.95-0.98)^b^
South	0.91 (0.90-0.92)^b^	0.95 (0.93-0.96)^b^
West	0.57 (0.56-0.58)^b^	0.52 (0.51-0.53)^b^
Unknown	0.14 (0.09-0.23)^b^	0.20 (0.12-0.36)^b^
Comorbidity score (modified)		
0	1 [Reference]	1 [Reference]
1	0.93 (0.92-0.94)^b^	0.91 (0.90-0.92)^b^
≥2	0.81 (0.80-0.82)^b^	0.79 (0.77-0.80)^b^
Mental health or substance abuse disorder	0.83 (0.82-0.85)^b^	0.80 (0.78-0.82)^b^
Outpatient visits 12 mos prior to index test, No.		
0-2	1 [Reference]	1 [Reference]
3-5	1.37 (1.35-1.39)^b^	1.39 (1.37-1.41)^b^
>6	1.67 (1.64-1.69)^b^	1.71 (1.68-1.73)^b^
Sexually transmitted disease 12 mos prior to test, No.	0.99 (0.81-1.20)	0.82 (0.66-1.03)
Pregnancy 12 mos prior to test	0.78 (0.76-0.80)^b^	0.76 (0.74-0.79)^b^
Gynecological symptoms within 30 d of test	0.76 (0.74-0.77)^b^	0.75 (0.73-0.77)^b^
Sexually transmitted disease testing 12 mos after test	0.497 (0.489-0.504)^b^	0.61 (0.60-0.62)^b^
Family planning within 30 d of test	1.45 (1.41-1.48)^b^	1.37 (1.33-1.41)^b^

Abbreviations: aOR, adjusted odds ratio; HPV, human papillomavirus.

^a^ Years after interval cervical cancer screening test. Year 2: 12-24 months, year 3: 24-36 months.

^b^ *P* < .05.

## Discussion

The findings of this cohort study suggest that among commercially insured women with average risk who underwent cervical cancer screening in 2013 to 2014, cervical cancer screening tests were frequently overused. Within our cohort, nearly two-thirds of women underwent repeat screening within 3 years of their index test. Overtesting was particularly common in younger women and in women screened with cytologic testing alone. Women who did not undergo repeat cervical cancer screening were less likely to receive a gynecological examination.

Overuse of cervical cancer screening tests is common.^16,18,19,20,21^ A single-center study by Almeida et al^18^ reported overscreening in 45% of the cohort, while a statewide analysis of Medicaid beneficiaries in Pennsylvania by Parekh et al^16^ found the 65% of women aged older than 30 years were screened too frequently in 2009 to 2010. Similarly, patient survey data have suggested that a large number of women undergo testing more frequently than recommended, while surveys of physicians also commonly report performance of screening tests more frequently than indicated.^19,20,21,22,23^ In addition to testing too frequently, performance of cervical cancer screening tests in women younger than 21 years or older than 65 years and women who have undergone hysterectomy, all of whom are at low risk, is common.^11,12,13,14^ In our cohort, two-thirds of women were overscreened. As current guidelines recommend screening no more often than every 5 years in women who have undergone HPV testing, our findings would likely have been even more pronounced if follow-up were extended beyond 36 months.

Overuse of cervical cancer screening leads to a number of downstream consequences.^24^ Identification of transient HPV infections and low-grade abnormal findings likely to resolve spontaneously often leads to further diagnostic testing with colposcopy and biopsies and, possibly, ablative or excisional procedures.^24^ These procedures are often associated with psychological stress and physical symptoms.^4,24^ Furthermore, treatment of preinvasive cervical lesions is associated with adverse pregnancy outcomes, including preterm birth.^24,25^ Additionally, overuse of these screening tests and diagnostic procedures have a significant financial impact for the health care system.

A number of factors likely influence overuse of cervical cancer screening tests. As data on testing strategies evolve, guidelines change frequently, and multiple several professional societies release their own, different screening guidelines, patients and clinicians are often unaware of screening recommendations or may be confused by the lack of consensus among guidelines.^9,26^ Furthermore, women are often uncertain of when they last underwent cervical cancer screening, which may result in unnecessary testing.^27^ Even if women are aware of screening guidelines and their history, they often prefer to continue annual cervical cancer screening.^26^ Similarly, clinicians often believe that women are uncomfortable with extended intervals between screening tests, fear that less frequent screening will result in disengagement from gynecologic services, and are concerned about medical liability.^9,21,28,29^ There is currently minimal financial disincentive for clinicians to perform more frequent testing.

We noted that women who did not undergo cervical cancer screening were substantially less likely to have a gynecologic examination in that year. The value of screening pelvic examinations in women who are asymptomatic has long been debated.^30,31,32^ The American College of Obstetricians and Gynecologists^30^ recommends shared decision-making to guide performance of examination. In contrast, the American College of Physicians^32^ recommends against routine pelvic examination, while the US Preventive Services Task Force^31^ concluded that the available data were insufficient for a recommendation. Our data indicate that women who do not undergo cervical cancer screening in a given year are substantially less likely to undergo a routine gynecological examination. These findings suggest that women may disengage from gynecologic care if they do not require cervical cancer screening. Alternatively, given the ease of cervical cancer screening, physicians may reflexively perform testing in women who are presenting for their annual gynecological examination. Disengagement from follow-up has the potential to result in decreased follow-up for appropriate screening in future years.

### Limitations

While our study benefits from the inclusion of a large cohort of women from across the US, it also has some important limitations. First, we cannot exclude the possibility that a small number of screening tests were performed as follow-up for abnormal findings and not for primary screening. However, this is likely a very small number of tests, since we excluded women with any diagnostic coding for even mild pathological findings, as well as women who underwent any diagnostic or treatment-related procedures for cervical dysplasia. Similarly, we may have undercaptured pelvic examinations in women who had their encounters billed as visits for gynecological conditions. However, the intent of our analysis was to capture screening examinations in asymptomatic women, not those undergoing evaluations for symptomatic gynecological disease. Third, our study includes only women with commercial insurance, and these findings may not be generalizable to other populations, including older and younger women. Fourth, our analysis is based on recommendations from the American Cancer Society 2012 guidelines. Numerous organizations have developed and reported varying guidelines over time, and our findings may differ slightly based on other recommendations. Fourth, our analysis examined data from 2013 and 2014, and these data may not necessarily reflect current screening patterns. Fifth, as with any observational study, we are unable to capture unmeasured factors that may have influenced testing, such as symptoms or abnormal findings on physical examination.

## Conclusions

The findings of this cohort study suggest that overuse of cervical cancer screening was common at the population level, with nearly two-thirds of women undergoing unnecessary screening within 3 years of index testing. From a policy standpoint, interventions to mitigate overuse of cervical cancer screening are of great interest. Prior studies have examined electronic health record interventions, including automated alerts and links to guidelines, clinician education, and provision of manual pocket guides.^33^ While these interventions have demonstrated modest short-term success, none of these interventions were randomized or compared with contemporaneous controls, and most interventions focused on decreasing early initiation of Papanicolaou testing in young women.^33^ Evaluation of interventions to align clinical practice with evidence-based guidelines for cervical cancer screening are clearly needed to help reduce patient harm and medical waste.

## Notes

Supplement. **eTable 1.** Diagnostic Coding for the Analysis **eTable 2.** Performance of Repeat Cervical Cancer Screening After Index Testing Stratified by Age
