# Supplementary material for: Overuse of Cervical Cancer Screening Tests Among Women With Average Risk in the United States From 2013 to 2014
Source: JAMA Netw Open. 2021 Apr 29;4(4):e218373. doi: 10.1001/jamanetworkopen.2021.8373 (PMC8085723; doi:10.1001/jamanetworkopen.2021.8373)
Supplement: Supplement. — eTable 1. Diagnostic Coding for the Analysis eTable 2. Performance of Repeat Cervical Cancer Screening After Index Testing Stratified by Age [file jamanetwopen-e218373-s001.pdf]

Supplemental Online Content

Wright JD, Chen L, Tergas AI, et al. Overuse of cervical cancer screening tests among women with average risk in the United States from 2013 to 2014. *JAMA Netw Open*. 2021;4(4):e218373. doi:10.1001/jamanetworkopen.2021.8373

eTable 1. Diagnostic Coding for the Analysis

eTable 2. Performance of Repeat Cervical Cancer Screening After Index Testing Stratified by Age

This supplemental material has been provided by the authors to give readers additional information about their work.

© 2021 Wright JD et al. *JAMA Network Open.*

eTable 1. Diagnostic Coding for the Analysis

**ICD-9**	**ICD-10**	**CPT/HCPCS**
Cytology		
Procedure:<br>91.46		CPT:<br>88141, 88142, 88143, 88144, 88145, 88147, 88148, 88150, 88152, 88153, 88154, 88155, 88164, 88165, 88166, 88167, 88174, 88175;<br>HCPCS:<br>G0123, G0143, G0144, G0145, G0147, G0148, P3000, G0124, G0141, P3001, Q0091, G0101
HPV		
Diagnosis:<br>V73.81.	Diagnosis:<br>Z11.51.	CPT:<br>87620, 87621, 87622, 87623, 87624, 87625, 0500T;<br>HCPCS:<br>G0476.
Gynecologic examination		
Diagnosis:<br>V72.31, V72.32, V76.2, V76.47, V76.49.	Diagnosis:<br>Z01.411, Z01.419, Z01.42, Z12.4, Z12.72, Z12.79, Z12.89.	
Gynecologic cancer		
Diagnosis:<br>179.*, 180.*, 182.*, 183.*, 158.8, 158.9.	Diagnosis:<br>C53.*-C57.*, C48.1, C48.2, C48.8, R87.614.	
Abnormal histology or cytology, positive HPV test, or unsatisfactory cytology		
Diagnosis:<br>795.00, 795.01, 795.02,795.03, 795.04, 795.05, 795.07, 795.08, 795.09, 795.10, 795.11, 795.12, 795.13, 795.14, 795.16, 795.18, 795.19, 622.10, 622.11, 622.12, 623.0, 233.1, 233.30, 233.31, 233.39,	Diagnosis:<br>R87.610, R87.611, R87.612, R87.613, R87.615, R87.616, R87.618, R87.619, R87.620, R87.621, R87.622, R87.623, R87.624, R87.625, R87.628, R87.629, R87.69, N87.0, N87.1, N87.9, N89.3, D06.0, D06.1, D06.7, D06.9, D07.2, D07.30, D07.39,	
Gynecologic procedures (colposcopy, biopsy, conization and cauterization)		
Procedure:<br>70.12, 67.11, 67.12, 67.2, 67.32, 67.33.	Procedure:<br>0UJD7ZZ, 0UJD8ZZ, 0UJH7ZZ, 0UJH8ZZ 0UBC7ZZ, 0UBC8ZZ, 0UCC7ZZ, 0UCC8ZZ 0U5C7ZZ, 0U5C8ZZ.	CPT:<br>57420, 57452, 57421, 57455, 57500, 57505, 57454, 57456, 57450, 57460, 57461, 57520, 57522, 57510, 57511, 57513.
Diethylstilbestrol exposure		
Diagnosis:		

© 2021 Wright JD et al. *JAMA Network Open.*

646.80, 760.76, V13.29, V15.89		
HIV infection		
Diagnosis:<br>042, 043.1, 043.2, 043.3, 043.9, 044.0, 044.9, 079.53, 647.60, 647.61,<br>647.63, V08, V12.09.	Diagnosis:<br>B20.*, B21.*, B22.*, B23.*, B24.*, B97.35, O98.5*, O98.7*, Z21, Z86.19.	
Leukemia		
204.*, 205.*, 206.*, 207.*, 208.*.	Diagnosis:<br>C91.*, C92.*, C93.*, C94.*, C95.*.	
Neutropenia		
Diagnosis:<br>288.0*, 289.53, 780.61.	Diagnosis:<br>D70.*, D73.81, R50.81.	
Organ transplantation		
Diagnosis:<br>996.8*, V42.9.	Diagnosis:<br>T86.*, Z94.9.	
Hysterectomy		
Procedure:<br>68.3, 68.39, 68.4, 68.49, 68.6, 68.69, 68.9;<br>68.31, 68.41, 68.61;<br>68.51, 68.71; 68.5, 68.59, 68.7, 68.79.	Procedure:<br>0UT90*; 0UT94*, 0UT98*, 0UT9F*; 0UT97*;	CPT:<br>58150, 58152, 58180, 58200, 58210, 58950, 58951, 58953, 58954, 58956;<br>58541, 58542, 58543, 58544, 58548, 58550, 58552, 58553, 58554, 58570, 58571, 58572, 58573;<br>58260, 58262, 58263, 58267, 58270, 58275, 58280, 58285, 58290, 58291, 58292, 58293, 58294.
Immunosuppressants		
NDC:<br>azathioprine, 6-mercaptopurine, methotrexate, etanercept, tacrolimus, sirolimus, infliximab, adalimumab, muromonab-CD3, basiliximab, daclizumab, immune globulin, cyclophosphamide, cyclosporine, anakinra, mycophenolate mofetil, and other immunosuppressants.		
Outpatient visits		
Place of service:<br>11 Office; 49 Independent clinic; 50 Federally qualified health care center; 71 Public health clinic; 72 Rural health clinic.		
STI history		
Diagnosis:<br>079.88, 079.98, 098.0, 098.10, 098.15, 098.16, 098.17, 098.19, 098.2, 098.30,	Diagnosis:<br>A54.00, A54.02, A54.03, A54.09, A54.24, A54.29, A54.85, A54.86, A54.89, A54.9, A56.00, A56.01, A56.02, A56.09, A56.11, A56.19, A56.2, A56.8,	

© 2021 Wright JD et al. *JAMA Network Open.*

098.35, 098.36, 098.37, 098.39, 098.86, 098.89,<br>099.50, 099.53, 099.54, 099.55, 099.56, 099.59, 099.8, 099.9, 647.10, 647.11, 647.13 647.20, 647.21, 647.23, 647.60, 647.61, 647.63,<br>V02.7, V02.8.	A63.0, A63.8, A64, A74.81, A74.89, A74.9, O98.211, O98.212, O98.213, O98.219, O98.311, O98.312, O98.313, O98.319, O98.511, O98.512, O98.513, O98.519, Z22.4.	
Mental health and substance abuse		
Diagnosis:<br>290.*-319.*.	Diagnosis:<br>F01.*-F99.*.	
Gynecologic symptoms		
Diagnosis:<br>041.9, 112.1, 131.01, 616.10, 623.5, 623.8, 625.8, 646.60, 646.61, 646.63, 646.80, 646.81, 646.83.	Diagnosis:<br>A59.01, B37.3, B96.89, N76.0, N76.1, N76.2, N76.3, N89.4, N89.8, N94.89, O23.511, O23.512, O23.513, O23.519, O23.521, O23.522, O23.523, O23.529, O23.591, O23.592, O23.593, O23.599, O23.90, O23.91, O23.92, O23.93, O99.89.	
STI testing		
Diagnosis:<br>V01.6, V73.88, V73.89, V73.98, V74.5.	Diagnosis:<br>Z11.3, Z11.4, Z11.59, Z11.8, Z11.9, Z20.2, Z20.6.	CPT:<br>86592, 86593, 86631, 86632, 86689, 86701, 86702, 86703, 86780, 87110, 87270, 87320, 87389, 87390, 87391, 87490, 87491, 87492, 87534, 87535, 87536, 87537, 87538, 87539, 87590, 87591, 87592, 87800, 87801, 87810, 87850;<br>HCPCS:<br>G0432, G0433, G0435.
Family planning		
Diagnosis:<br>V25.01, V25.02, V25.03, V25.04, V25.09, V25.11, V25.13, V25.2, V25.40, V25.41, V25.42, V25.43, V25.49, V25.5, V25.8, V25.9.	Diagnosis:<br>Z30.011, Z30.012, Z30.013, Z30.014, Z30.015, Z30.016, Z30.017, Z30.018, Z30.019, Z30.02, Z30.09, Z30.2, Z30.40, Z30.41, Z30.42, Z30.43, Z30.430, Z30.431, Z30.433, Z30.44, Z30.45, Z30.46, Z30.49, Z30.8, Z30.9.	CPT:<br>11975, 11977, 57170, 58300;<br>HCPCS:<br>A4266, A4269, J1055, J7296, J7297, J7298, J7300, J7301, J7302, J7303, J7304, J7306, J7307, S4993.
Pregnancy		
Diagnosis:<br>630.*-679.*, V22.*, V23.*, V24.*, V27.*, V28.*;	-	CPT:<br>59400, 59409, 59410, 59425, 59426, 59430, 59510, 59514, 59515, 59610, 59612, 59614,

© 2021 Wright JD et al. *JAMA Network Open.*

Procedure:<br>73.51, 73.59, 74.0, 74.1, 74.2, 74.3, 74.4, 74.91, 74.99, 88.78.		59618, 59620, 59622, 76801, 76805, 76811, 76813, 76815, 76816, 76817, 76818, 76819, 76820, 76821, 76825, 76826, 76827, 76828, 99500, 99501, 0500F, 0501F, 0502F, 0503F;<br>HCPCS:<br>H1000, H1001, H1002, H1003, H1004, H1005.

© 2021 Wright JD et al. *JAMA Network Open.*

eTable 2. Performance of Repeat Cervical Cancer Screening After Index Testing Stratified by Age

	**Year 1**		**Year 2**		**Year 3**	
	**N**	**(%)**	**N**	**(%)**	**N**	**(%)**
30-39 years of age						
Second test in 12, 24, or 36 months	39,044	(6.5)	194,165	(52.0)	194,665	(67.8)
Time from index test, in months, median (IQR)	10.3	(8.1-11.7)	13.4	(12.4-16.3)	14.7	(12.5-23.2)
Type of second test						
Cotesting	16,612	(42.6)	74,048	(38.1)	79,052	(40.6)
Cytology	21,965	(56.3)	118,826	(61.2)	114,132	(58.6)
HPV testing	467	(1.2)	1,291	(0.7)	1,481	(0.8)
40-49 years of age						
Second test in 12, 24, or 36 months	29,430	(4.0)	240,485	(50.5)	249,088	(65.7)
Time from index test, in months, median (IQR)	11.4	(9.6-11.9)	13.3	(12.4-16.1)	14.5	(12.6-23.1)
Type of second test						
Cotesting	12,578	(42.7)	89,595	(37.3)	98,220	(39.4)
Cytology	16,451	(55.9)	149,423	(62.1)	149,130	(59.9)
HPV testing	401	(1.4)	1,467	(0.6)	1,738	(0.7)
50-59 years of age						
Second test in 12, 24, or 36 months	26,169	(3.5)	238,188	(47.4)	256,550	(62.3)
Time from index test, in months, median (IQR)	11.6	(10.2-12.0)	13.1	(12.4-15.6)	14.2	(12.5-23.3)
Type of second test						
Cotesting	9,730	(37.2)	78,072	(32.8)	89,625	(34.9)
Cytology	16,131	(61.6)	158,791	(66.7)	165,296	(64.4)
HPV testing	308	(1.2)	1,325	(0.6)	1,629	(0.6)
60-64 years of age						
Second test in 12, 24, or 36 months	6,495	(3.2)	47,692	(45.4)	32,198	(60.2)
Time from index test, in months, median (IQR)	11.6	(10.3-12.0)	13.0	(12.4-15.3)	14.0	(12.4-23.3)
Type of second test						
Cotesting	2,168	(33.4)	13,887	(29.1)	10,135	(31.5)
Cytology	4,246	(65.4)	33,553	(70.4)	21,880	(68.0)
HPV testing	81	(1.3)	252	(0.5)	183	(0.6)

© 2021 Wright JD et al. *JAMA Network Open.*
